# Exploratory study on the correlation between 14 lung cancer-related gene expression and specific clinical characteristics of NSCLC patients

**DOI:** 10.3892/mco.2013.153

**Published:** 2013-07-23

**Authors:** YI HAN, GUO LI, CHONGYU SU, HUA REN, XIANGYANG CHU, QIUYUE ZHAO, YANJUN ZHU, ZITONG WANG, BIN HU, GUANGYU AN, JINGBO KANG, WEI WANG, DAPING YU, XIAOYUN SONG, NING XIAO, YUNSONG LI, XIA LI, HUIYI YANG, GANG YU, ZHIDONG LIU

**Affiliations:** 1 Department of Thoracic Surgery, Beijing Chest Hospital, Beijing 101149, P.R. China; 2 Department of Thoracic Surgery, Chinese PLA General Hospital, Beijing 100853, P.R. China; 3 General Hospital of the Chinese Armed Police Forces, Beijing 100039, P.R. China; 4 General Hospital of the Air Force, PLA, Beijing 100142, P.R. China; 5 Beijing Chao-Yang Hospital, Capital Medical University, Beijing 100020, P.R. China; 6 Navy General Hospital, Beijing 100048, P.R. China; 7 SurExam Bio-Tech Co. Ltd., Guangzhou Technology Innovation Base, Science City, Guangzhou 510663, P.R. China

**Keywords:** non-small cell lung cancer, clinicopathological, expression of genes

## Abstract

Personalized medicine has become essential in the treatment of lung cancer. However, the lung cancer-related gene expression profiles in non-small cell lung cancer (NSCLC) patients have not been elucidated. In this study, the correlation between gene expression profiles and clinicopathological characteristics was investigated in NSCLC patients. A total of 95 patients were enrolled in this study. The mRNA expression levels of 14 genes were assessed by multiplex branched DNA liquidchip (MBL) technology and data on 9 clinicopathological characteristics of patients were collected simultaneously. The correlation between gene expression and clinicopathological characteristics was investigated. Out of the 9 clinicopathological parameters, 6 were associated with several of the 14 genes analyzed. Patient gender was associated with *TYMS* and *TOP2A*. Clinical stage was associated with *VEGFR2*, *KIT* and *HER2*. There was weak correlation between primary tumor size of ≤3 cm and the expression level of *KIT*. The mRNA expression levels of *VEGFR2* and *HER2* correlated with distant metastasis. *BRCA1*, *TYMS*, *TOP2A* and *HER2* were associated with histological type. Smoking correlated with higher expression levels of *BRCA1*, *TYMS* and *TOP2A* and lower expression levels of *PDGFRβ*. The results were suggestive of correlation between the clinicopathological parameters of the NSCLC patients and the mRNA expression levels of certain lung cancer-related genes, including *BRCA1*, *TYMS*, *TOP2A*, *PDGFRβ*, *VEGFR2*, *KIT* and *HER2*.

## Introduction

Lung cancer is a leading cause of mortality worldwide and non small-cell lung cancer (NSCLC) constitutes 80% of all lung cancer cases (1,2). NSCLC is commonly diagnosed at an advanced stage, which leads to a poor prognosis (3). More effective individualized cancer therapies are necessary to achieve the best possible outcome for each patient, which is the ultimate goal of personalized medicine (4). Studies were previoiusly conducted to identify genes related to treatment responses of NSCLC, breast, colorectal and other types of cancer exhibiting a high prevalence (5–7). The treatment of NSCLC may be significantly improved with improved understanding of the molecular basis of the disease and innovative laboratory technology. In this study, 14 lung cancer-related genes were selected, based on previous studies, and we attempted to identify the association between the genes mRNA expression levels and specific clinicopathological characteristics of NSCLC patients. This study may help develop novel ways of selection of the appropriate treatment strategy for patients with NSCLC using molecular diagnostic tools.

## Materials and methods

### Patient and specimen collection

A total of 142 Chinese NSCLC patients who underwent surgery at Beijing Chest Hospital, Capital Medical University, between May, 2010 and December, 2011 were screened. Of these 142 patients, 95 were enrolled into analysis according to rank randomization tests and SPSS software version 18.0 (SPSS Inc., Chicago, IL, USA) was used to randomly assign the observations. The surgery specimens were collected for detection of mRNA expression. Detection of the following genes was performed in this study: *ERCC1*, *BRCA1*, *TYMS*, *RRM1*, *TUBB3*, *STMN1*, *TOP2A*, *EGFR*, *PDGFRβ*, *VEGFR1*, *VEGFR2*, *KIT*, *HER2* and *IGF1R*. Subsequently, 9 patient clinicopathological parameters were assessed, including gender, age, clinical stage, primary tumor size, nodal status, distant metastasis status, histological type, differentiation and smoking history.

### Detection of mRNA expression levels

A novel multiplex branched DNA liquidchip (MBL) technology, which integrates the branched DNA (bDNA) and liquidchip technology, was developed for quantitative measurement of the gene mRNA levels in the formalin-fixed paraffin-embedded (FFPE) slides. MBL is a non-PCR-based technology. It is a nucleic acid sandwich hybridization platform in which targets are captured through cooperative hybridization of multiple probes and then conjunct with a fluorescence signal amplification system (8,9).

The MBL method was as follows: Sample pretreatment: The FFPE tissue samples were homogenized in a mixture of homogenizing solution at 65°C for 2 h. The homogenate was centrifuged to remove residual paraffin and debris and the supernatant was transferred to a fresh centrifuge tube. The homogenate (40 μl) was added to each well of a 96-well plate containing the reagents: 18.5 μl RNase-free water, 33.3 μl lysozyme solution, 2 μl blocking reagent, 1 μl capture beads and 5 μl target gene-specific pretreatment probe set. the 96-well plate was sealed and incubated at 54°C for 18 h on a shaker at 314 × g. The hybridization mixture was then transferred to a filtered 96-well plate. The unbound RNA and other debris in the wells were removed by washing three times with 250 μl wash buffer under a magnetic adsorption system.

Signals for bound target mRNA were developed using the following steps: incubation in 100 μl preamplifier solution at 50°C for 1 h; washing twice with 200 μl wash buffer; incubation in 100 μl amplifier solution at 50°C for 1 h; washing twice with 200 μl wash buffer; incubation in 100 μl labeled probe at 50°C for 1 h; and washing twice with 200 μl wash buffer. The samples were then developed with 100 μl streptavidin-phycoerythrin solution at 25°C for 30 min. The fluorescence value of each sample was analyzed using the Luminex 200 system (Luminex, Austin, TX, USA).

This MBL platform is currently used in China for clinical pharmacogenomic diagnosis in personalized chemotherapy for NSCLC in order to measure mRNA levels of *ERCC1*, *BRCA1*, *TUBB3* and *STMN1,* as well as *TYMS*, *RRM1*, *TOP2A*, *EGFR*, *PDGFR*, *VEGFR1*, *VEGFR2*, *KIT*, *HER2* and *IGF1R*.

### Statistical analysis

SPSS statistical software version 18.0 was used to analyze data in this study. For each gene, we divided the detection values into two groups according to the median value of the 95 patients. Detection values above the median constituted the high-level group, whereas values below the median constituted the low-level group. The χ^2^ test was used to assess the association between gene expression grades and each of the clinicopathological characteristics. The relevance of the two factors was revealed as the Spearman’s rank correlation and the correlation intensity was described as the correlation coefficient. All tests were two-sided and P<0.05 was considered to indicate a statistically significant difference.

## Results

### Patient characteristics

Of the 95 patients, 53 were males and 42 females. Sixty-one patients were <65 years and 34 were >65 years old. A total of 20, 18, 30 and 23 patients had clinical stage I, II, III and IV disease, respectively, whereas in 4 patients the clinical stage was undetermined. Distant metastasis was present in 21 patients, absent in 68 and undetermined in 6. Sixty-six patients had adenocarcinoma, 28 had non-adenocarcinoma and information on 1 patient was not available. The degree of differentiation was low, moderate, high and undetermined in 37, 30, 15 and 13 patients, respectively. Forty-nine patients were smokers, 42 had never been smokers and the smoking status of 4 was undetermined. The patient characteristics are listed in Table I.

### Gene expression profiles

The median mRNA expression level of *ERCC1* was 0.621 (range, 0.526–0.879) in the 95 patients. The 25% of the 95 detection values was 0.526, whereas the 75% was 0.879. The Q_U_-Q_L_ of the *ERCC1* mRNA expression was 0.353. The expression levels of the 14 genes are presented in detail in Table II.

### Correlation of gene expression levels and patient clinicopathological characteristics

Of the 14 genes, 6 (*ERCC1*, *RRM1*, *TUBB3*, *EGFR*, *VEGFR1* and *IGF1R*) exhibited no association with the nine clinicopathological characteristics. Furthermore, 3 patient characteristics (age, nodal status and differentiation) were not associated with any of the 14 genes assessed in this study.

*TYMS* and *TOP2A* correlated with gender. Male patients exhibited higher expression levels of *TYMS* (P=0.005) and *TOP2A* (P=0.017). Patients in the advanced stages of the disease were associated with higher levels of *VEGFR2* (P=0.028) and lower levels of *KIT* (P=0.016). In primary tumors, higher levels of *KIT* (P=0.016) were observed in patients with tumor size ≤3 cm (Table III). Among all NSCLC patients, 35% presented with negative nodal status. The expression levels of *VEGFR2* and *HER2* exhibited a direct correlation with distant metastasis. The correlation coefficients of *VEGFR2* and *HER2* were 0.285 (P=0.007) and 0.217 (P=0.041), respectively (Table IV).

The expression levels of *BRCA1*, *TYMS*, *TOP2A* and *HER2* were associated with histological type and the expression levels of *BRCA1*, *TYMS* and *TOP2A* were different between adenocarcinoma and non-adenocarcinoma patients (the correlation coefficients of the three genes were 0.419, P=0.000; 0.293, P=0.004; and 0.247, P=0.017, respectively). There was a marked correlation between the expression levels of *BRCA1* and histological type. However, the expression levels of *HER2* were inversely associated with histological type. The correlation coefficient was 0.265 (P=0.010) (Table IV). This finding indicates that patients with adenocarcinoma exhibit low expression levels of *HER2*.

Similarly, *BRCA1*, *TYMS*, *TOP2A* and *PDGFRβ* were associated with smoking status (P=0.009, P=0.015, P=0.004 and P=0.026, respectively). Smoking patients tended to exhibit higher expression levels of *BRCA1*, *TYMS* and *TOP2A* (the correlation coefficients were 0.275, P=0.008; 0.254, P=0.015; and 0.298, P=0.004, respectively) and lower expression levels of *PDGFRβ* (correlation coefficient: −0.234, P=0.026) (Table IV).

## Discussion

In this study, we assessed the mRNA expression levels of 14 genes and investigated the association between gene expression levels and patient characteristics. Of the 9 clinicopathological parameters, six were associated with some of the 14 genes analyzed. Patient gender was associated with *TYMS* and *TOP2A*. Clinical stage was associated with *VEGFR2*, *KIT* and *HER2*. There was a weak correlation between a primary tumor size of ≤3 cm and the expression level of *KIT*. The mRNA expression levels of *VEGFR2* and *HER2* correlated with distant metastasis. *BRCA1*, *TYMS*, *TOP2A* and *HER2* were associated with histological type. Smoking correlated with higher expression levels of *BRCA1*, *TYMS* and *TOP2A* and lower expression levels of *PDGFRβ*.

Genes are critical elements in the physiology of cancer, including DNA synthesis, DNA repair and mitosis. Gene expression levels are associated with a response to chemotherapy and targeted therapies (5,10). Traditionally, treatment selection has been based on clinicopathological characteristics and imaging techniques, including computed tomography (CT) or magnetic resonance imaging (MRI).

In previous studies, different mRNA expression levels of genes were associated with different responses to chemotherapy. Olaussen *et al* observed that the expression level of *ERCC1* positively correlated with the response to cisplatin-based and neoadjuvant chemotherapy in NSCLC patients (11). Overexpression of *BRCA1* was strongly associated with poor survival in NSCLC patients. The mRNA expression levels of *BRCA1* have been previously associated with differential sensitivity to cisplatin and antimicrotubule drugs (12). The association analysis in this study revealed that patients with adenocarcinoma and smokers were more likely to exhibit higher mRNA expression levels of *BRCA1*. Therefore, it was hypothesized that smoking patients with adenocarcinoma would not benefit from cisplatin and antimicrotubule therapies. However, larger studies are required to reach a definitive conclusion. A previous study demonstrated that males had higher tumor expression levels of *BRCA1* compared to females (P=0.020) (13). We did not observe this difference in the present study, which may be attributed to diversities in race and ethnicity. However, histological type and smoking status may be critical for the treatment of NSCLC.

Lung cancer patients with high expression levels of *TYMS* mRNA were associated with resistance to fluorouracil-based chemotherapy (14). Ceppi *et al* reported that Caucasian patients with squamous cell carcinoma exhibited higher TYMS protein expression levels (15). By contrast, another study conducted on Japanese patients demonstrated that tumor cells with higher *TYMS* expression exhibit higher proliferative activity in NSCLC, especially adenocarcinoma (16). We observed that patients with adenocarcinoma exhibited higher *TYMS* levels compared to those with squamous cell carcinoma, which was in accordance with the results of the latter study (15). The differrences in the results among studies may be partly due to the racial and ethnic diversities. However, this observation requires elucidation through further large-sample studies. Smokers tended to exhibit higher expression levels of *TYMS*. This was in accordance with previous studies. Since gene expression levels were found to be associated with clinicopathological characteristics, they may be useful in the selection of NSCLC patients who may benefit from treatment with *TYMS*-inhibiting agents.

A study demonstrated that low expression levels of *RRM1* were a favorable indicator for tumor response to gemcitabine, whereas high levels were associated with drug resistance (17). Low expression levels of *TUBB3* was a favorable indicator for tumor response to paclitaxel and vinblastine, whereas high levels were associated with drug resistance (18). Overexpression of the *EGFR* gene was associated with improved response to erlotinib or gefitinib therapy and increased survival in NSCLC patients (19).

*STMN1* was characterized as an important regulatory gene of microtubule dynamics, which were comprehensively studied in cancer (20). *TOP2A* and *HER2* were extensively studied in breast cancer. Previous studies reported that *TOP2A* amplification is a marker of sensitivity to anthracyclines (21,22). *TOP2A* catalyzes the relaxation of supercoiled DNA and is associated with DNA proliferation and repair (23,24). Previously, a correlation with positive staining for *TOP2A* was identified in male adenocarcinoma patients (25). In this study, we observed similar results regarding the association between expression data and clinicopathological characteristics. Furthermore, we found that the expression of *TOP2A* was associated with patient smoking status. Since *TOP2A* amplification is a marker of sensitivity to anthracyclines, smoking male patients with adenocarcinoma are likely to benefit from anthracycline treatment.

Trastuzumab therapy may be used as a general paradigm for successful targeted therapy: a positive *HER2* status determines the indication for trastuzumab therapy and it is important to use simple, accurate, widely applicable and reproducible methods to screen tumors for gene amplification and/or overexpression (26). Nakamura *et al* reported that overexpression of *HER2* was detected in adenocarcinoma more frequently compared to squamous cell carcinoma in Japanese NSCLC patients (27). Our results demonstrated the opposite; however, these results require further validation. It has been proven that *HER2* expression, particularly in adenocarcinoma, is associated with a poor prognosis (28,29). Studies investigating the expression of *HER2* in advanced or metastatic NSCLC patients are required to verify our results, which suggest that the expression of *HER2* is associated with metastasis in NSCLC patients. This would be a novel research direction.

*KIT* is a proto-oncogene encoding KIT protein, a typical type III tyrosine kinase receptor. Clinical observations demonstrated that the positive rate of *KIT* expression was 87–100% (30) in gastrointestinal stromal tumors (GISTs) and that high *KIT* expression levels are associated with the response to imatinib. *PDGFRβ* is a single transmembrane glycoprotein and a member of the type III protein tyrosine kinase family, which plays an important role in tumor cell proliferation and angiogenesis (31). Sunitinib and sorafenib are *PDGFRβ* tyrosine kinase inhibitors in wide clinical application that directly inhibit tumor cell proliferation and angiogenesis (32). The number of studies focusing on the mRNA expression level of *PDGFRβ* in NSCLC, particularly on the association between expression and clinicopathological characteristics, is limited. It has been proven that higher levels of *PDGFRβ* expression were associated with higher levels of *VEGF* expression. Therefore, *PDGFRβ*-overexpressing tumors may be effectively treated with imatinib (33). In this study, we observed that NSCLC patients who were smokers tended to exhibit lower expression levels of *PDGFRβ*. This finding suggests that *PDGFRβ* is a novel biomarker in NSCLC and sets a new direction for future studies.

VEGF is a potent growth factor for endothelial cells. It binds to its receptors, VEGFR-1, VEGFR-2 and, to a lesser extent, VEGFR-3, causing proliferation and migration of endothelial cells, thus promoting angiogenesis (34). Although VEGF overexpression has been directly associated with the process of angiogenesis in NSCLC, the number of studies on clinicopathological characteristics is limited. A previous study focused on the correlation between VEGF levels and the response to treatment with bevacizumab (35).

It has been demonstrated that biomarkers are associated with NSCLC diagnosis and prognosis. However, the association between these genes and specific clinicopathological parameters has not been elucidated. Patient clinicopathological characteristics are critical in the selection of treatment regimens.

Unlike previous testing technology, such as immunohistochemistry (IHC) and fluorescence *in situ* hybridization (FISH), MBL technology was used in this study. MBL exhibits high sensitivity and parallel detection and is suitable for various sample types. MBL technology is a non-PCR-based technology at the molecular level and is widely used in clinical diagnosis (36,37).

In clinical practice, the elucidation of the association between gene expression profiles and clinicopathological characteristics may aid physicians in selecting patient-suitable treatments. Our findings confirmed an association between expression levels of 14 genes and clinicopathological parameters of NSCLC patients, which increases the possibility of including certain biomarkers in NSCLC diagnostics.

This study investigated the gene expression profiles in Chinese NSCLC patients and systematically analyzed the association of mRNA expression levels with patient characteristics. However, due to the limited sample size and lack of efficacy data, a closer look on the association between gene expression profiles and NSCLC treatment was not feasible. Therefore, additional studies are required, including larger patient samples and clinical outcome data.

In conclusion, this study demonstrated an association between the expression of certain lung cancer-related genes and the clinicopathological parameters of NSCLC patients, which may lead to the inclusion of biomarker detection, in combination with the traditional clinicopathological parameter assessment, in the selection of treatment for NSCLC patients. Furthermore, MBL technology was proven to be a feasible platform for clinical gene tests.

## Figures and Tables

**Table I:** Characteristics of NSCLC patients.

Characteristics	No. (%)
Gender	
Male	53 (55.8)
Female	42 (44.2)
Age (years)	
<65	61 (64.2)
≥65	34 (35.8)
Stage	
I	20 (21.1)
II	18 (18.9)
III	30 (31.6)
IV	23 (24.2)
Undetermined	4 (4.2)
Primary tumor	
T1	24 (25.3)
T2	37 (38.9)
T3	16 (16.8)
T4	12 (12.6)
Undetermined	6 (6.3)
Lymph node status	
N0	31 (32.6)
N1	17 (17.9)
N2	33 (34.7)
N3	8 (8.4)
Undetermined	6 (6.3)
Distant metastasis	
M0	68 (71.6)
M1	21 (22.1)
Undetermined	6 (6.3)
Histological type	
Adenocarcinoma	66 (69.5)
Non-adenocarcinoma	28 (29.5)
Undetermined	1 (1.1)
Differentiation	
Low	37 (38.9)
Moderate	30 (31.6)
High	15 (15.8)
Undetermined	13 (13.7)
Smoking history	
Never smoker	42 (44.2)
Smoker	49 (51.6)
Undetermined	4 (4.2)

NSCLC, non-small cell lung cancer.

**Table II:** Expression levels of genes in 95 NSCLC tumors.

Gene	Median value	Quartile		Interquartile range (Q_U_-Q_L_)

		25%	75%	
*ERCC1*	0.621	0.526	0.879	0.353
*BRCA1*	0.064	0.038	0.109	0.071
*TYMS*	0.107	0.059	0.205	0.146
*RRM1*	0.237	0.177	0.340	0.163
*TUBB3*	0.105	0.054	0.297	0.243
*STMN1*	1.500	1.096	2.637	1.541
*TOP2A*	0.160	0.082	0.348	0.266
*EGFR*	0.507	0.285	0.957	0.672
*PDGFR*	0.123	0.075	0.196	0.121
*VEGFR1*	0.067	0.048	0.109	0.061
*VEGFR2*	0.202	0.115	0.327	0.212
*KIT*	0.179	0.091	0.309	0.218
*HER2*	0.294	0.154	0.466	0.312
*IGF1R*	0.189	0.118	0.289	0.171

NSCLC, non-small cell lung cancer; *ERCC1*, excision repair cross-complementing rodent repair deficiency, complementation group 1; *BRCA1*, breast cancer 1; *TYMS*, thymidylate synthetase; *RRM1*, ribonucleotide reductase M1; *TUBB3*, tubulin, β 3 class III; *STMN1*, stathmin 1; *TOP2A*, topoisomerase II α; *EGFR*, epidermal growth factor receptor; *PDGFR*, platelet-derived growth factor receptor; *VEGFR1*, vascular endothelial growth factor receptor 1; *HER2*, human epidermal growth factor receptor 2; *IGF1R*, insulin-like growth factor 1 receptor; Q_U_, upper quartile; Q_L_, lower quartile.

**Table III:** Relationships between mRNA levels and clinical characteristics.

Characteristic	Statistics	*BRCA1*	*TYMS*	*STMN1*	*TOP2A*	*PDGFR*	*VEGFR2*	*KIT*	*HER2*
Gender	χ^2^	3.042	7.846	0.255	5.702	1.318	0.255	1.771	0.008
	P-value	0.081	0.005	0.614	0.017	0.251	0.614	0.183	0.927
Age (years)	χ^2^	0.124	0.006	0.006	0.006	3.176	0.006	3.200	0.608
	P-value	0.725	0.939	0.939	0.939	0.075	0.939	0.074	0.436
Stage	χ^2^	1.455	1.769	4.989	3.949	0.626	9.081	10.277	8.388
	P-value	0.693	0.622	0.173	0.267	0.890	0.028	0.016	0.039
Primary tumor	χ^2^	4.832	1.482	4.314	3.665	3.829	6.149	10.267	1.799
	P-value	0.185	0.686	0.230	0.300	0.281	0.105	0.016	0.615
Lymph nodes	χ^2^	3.692	7.394	0.839	5.162	3.622	0.353	1.385	1.073
	P-value	0.297	0.060	0.840	0.160	0.305	0.950	0.709	0.784
Distant metastasis	χ^2^	0.036	0.095	0.036	0.653	0.328	7.222	1.709	4.183
	P-value	0.849	0.758	0.849	0.419	0.567	0.007	0.191	0.041
Histological type	χ^2^	16.481	8.074	1.831	5.713	1.075	1.075	3.255	6.618
	P-value	0.000	0.004	0.176	0.017	0.300	0.300	0.071	0.010
Differentiation	χ^2^	1.462	5.942	5.544	0.795	0.443	0.443	4.043	5.427
	P-value	0.481	0.051	0.063	0.672	0.801	0.801	0.132	0.066
Smoking history	χ^2^	6.867	5.888	0.268	8.106	4.988	0.507	1.357	0.085
	P-value	0.009	0.015	0.605	0.004	0.026	0.476	0.244	0.771

Statistics were calculated by the χ^2^ test and the undetermined samples were not included. The cut-off point of the primary tumor was 3 cm. *BRCA1*, breast cancer 1; *TYMS*, thymidylate synthetase; *STMN1*, stathmin 1; *TOP2A*, topoisomerase II α; *VEGFR2*, vascular endothelial growth factor receptor 2; *PDGFR*, platelet-derived growth factor receptor; *HER2*, human epidermal growth factor receptor 2.

**Table IV:** Correlations between mRNA levels and clinical characteristics.

Characteristic	Statistics	*BRCA1*	*TYMS*	*STMN1*	*TOP2A*	*PDGFR*	*VEGFR2*	*KIT*	*HER2*
Gender	r	0.179	0.287	0.052	0.245	−0.118	0.052	−0.137	−0.009
	P-value	0.083	0.005	0.618	0.017	0.256	0.618	0.187	0.928
Age (years)	r	0.036	0.008	−0.008	0.008	−0.171	−0.008	0.184	−0.080
	P-value	0.729	0.940	0.940	0.940	0.076	0.940	0.075	0.441
Stage	ρ	−0.056	0.012	−0.061	0.014	0.082	0.276	−0.216	0.140
	P-value	0.595	0.912	0.567	0.896	0.442	0.008	0.040	0.186
Primary tumor	ρ	−0.036	0.044	0.149	0.151	−0.102	−0.018	−0.161	−0.011
	P-value	0.738	0.681	0.164	0.159	0.340	0.864	0.132	0.921
Lymph nodes	ρ	−0.023	0.220	0.042	0.197	0.060	0.043	−0.111	0.075
	P-value	0.833	0.039	0.693	0.064	0.577	0.687	0.300	0.483
Distant metastasis	r	0.020	0.033	0.020	0.086	0.061	0.285	−0.139	0.217
	P-value	0.851	0.761	0.851	0.425	0.572	0.007	0.195	0.041
Histological type	r	−0.419	−0.293	−0.140	−0.247	0.107	0.107	−0.186	0.265
	P-value	0.000	0.004	0.180	0.017	0.305	0.305	0.073	0.010
Differentiation	ρ	−0.123	−0.264	−0.260	−0.098	−0.069	0.069	0.144	0.115
	P-value	0.272	0.016	0.018	0.383	0.540	0.540	0.197	0.304
Smoking history	r	0.275	0.254	0.054	0.298	−0.234	0.075	−0.122	−0.031
	P-value	0.009	0.015	0.609	0.004	0.026	0.482	0.249	0.774

The undetermined samples were not included in statistics. The cut-off point of the primary tumor was 3 cm. r, Pearson’s product-moment correlation coefficient; ρ, Spearman’s rank correlation coefficient; *BRCA1*, breast cancer 1; *TYMS*, thymidylate synthetase; *STMN1*, stathmin 1; *TOP2A*, topoisomerase II α; *PDGFR*, platelet-derived growth factor receptor; *VEGFR2*, vascular endothelial growth factor receptor 2; *HER2*, human epidermal growth factor receptor 2.

## Abbreviations

ERCC1: excision repair cross-complementing rodent repair deficiency, complementation group 1
BRCA1: breast cancer 1
TYMS: thymidylate synthetase
RRM1: ribonucleotide reductase M1
TUBB3: tubulin, β 3 class III
STMN1: stathmin 1
TOP2A: topoisomerase II α
EGFR: epidermal growth factor receptor
VEGFR1: vascular endothelial growth factor receptor 1
HER2: human epidermal growth factor receptor 2
IGF1R: insulin-like growth factor 1 receptor
PDGFR: platelet-derived growth factor receptor
